# Modeling the Microsurgical Learning Curve Using a Poisson-Based Statistical Approach for Skill Assessment

**DOI:** 10.7759/cureus.83009

**Published:** 2025-04-25

**Authors:** Pablo J Villanueva, Hector I Rodriguez, Taku Sugiyama, Dara O'Keeffe, Guillermo Villanueva, Barbara M Villanueva, Adam F Roche

**Affiliations:** 1 Faculty of Medical Sciences, Microsurgical Laboratory, University of Buenos Aires, Buenos Aires, ARG; 2 Engineering, National University of Salta, Salta, ARG; 3 Neurosurgery, Hokkaido University, Sapporo, JPN; 4 Surgical Affairs, Royal College of Surgeons in Ireland (RCSI) University of Medicine and Health Sciences, Dublin, IRL; 5 Data Science, National University of Salta, Salta, ARG; 6 Royal College of Surgeons in Ireland (RCSI) SIM Center for Simulation Education and Research, RCSI University of Medicine and Health Sciences, Dublin, IRL

**Keywords:** microsurgery simulation, microsurgical skill assessment, microsurgical training, surgical education, surgical skill assessment

## Abstract

Objective: The learning curve (LC), a multifaceted concept, plays a pivotal role in evaluating surgical training. This study aimed to define critical inflection points in the microsurgical learning curve, develop a reliable index for skill assessment, and statistically validate this approach using Poisson distribution theory.

Method: A standardized microsurgical training protocol was employed using a biological simulator. Data regarding time to complete the task and error rates were collected over 132 attempts by a single operator. The primary outcome variable, the major mistake average (MMA), was used to generate a learning curve. Its progression was analyzed using autoregressive integrated moving average (ARIMA) modeling and validated with Poisson dispersion theory to determine the randomness of error occurrence at advanced stages of training. The entire trial was conducted by a single operator, a consultant neurosurgeon from our institution, who had been properly instructed on the protocol and the corresponding operator's manual.

Results: Task completion time (TCT) ranged from 860 to 3,054 seconds (mean: 1,472 seconds; R² = 0.561). MMA peaked at the 19th attempt (0.263) and decreased progressively, reaching 0.091 by the 132nd attempt (R² = 0.835). Three distinct phases of learning were identified, culminating in a plateau phase during which major mistakes followed a Poisson distribution (Chi² = 3.841), suggesting random occurrence independent of skill deficits.

Conclusion: The MMA was found to be a robust and objective indicator of microsurgical proficiency. Its statistical validation using Poisson distribution theory supports its utility in skill assessment and training programs. Further studies involving multiple operators are warranted to confirm these findings.

## Introduction

Acquiring technical skills and simulating realistic surgical scenarios are among the most challenging and essential components of surgical training. The integration of manual proficiency with theoretical knowledge is critical for enhancing operative performance and ensuring patient safety.

Numerous institutions and authors have contributed extensively to the development of structured protocols, objective methodologies, and rigorous data analysis tools aimed at skill evaluation in surgical education [1-11]. A fundamental goal in this field is the construction and interpretation of a precise learning curve (LC) [12].

The LC is inherently multifactorial. Although a comprehensive discussion of its theoretical foundations is beyond the scope of this manuscript, a brief overview is warranted. The concept dates back to 1885, when Hermann Ebbinghaus proposed the "Forgetting Curve." Since then, the learning curve has evolved across various disciplines, including education, psychology, and surgical sciences.

In surgical education, the LC is typically represented by a graphical depiction of a trainee’s performance over time. The performance is quantified through selected variables, such as task completion time or error rates, measured across multiple repetitions. These data points form a curve, the shape of which reflects the progression and stabilization of skills.

A rigorous LC analysis requires the selection of relevant variables, the use of reliable measurement instruments, and statistical modeling tailored to the field of application. This approach enables objective evaluations and facilitates meaningful comparisons.

In recent years, the field of surgical education has witnessed significant advancements in data science and simulation technologies. These innovations have improved the capacity to acquire and interpret performance data [13-15]. However, bibliometric analyses indicate that foundational principles, such as robust data collection and reliable interpretation frameworks, remain essential to accurate skill assessment [6,9,10].

The aims of the present study are as follows: 1) to apply a validated training protocol and data acquisition system to construct a solid microsurgical learning curve; 2) to propose and validate a reliable index for skill assessment; and 3) to statistically evidence the nature of our findings by using Poisson dispersion theory.

## Materials and methods

Study design

A previously validated microsurgical training protocol [1], utilizing a human placenta as a biological simulator, was implemented to assess skill acquisition in end-to-end arterial anastomosis. This model was chosen for its ability to accurately replicate essential aspects of microsurgical dissection and suturing in experimental/simulation scenarios.

To minimize variability and isolate the learning process, all procedures were performed by a single operator who had received prior instruction in micromovement techniques specific to this protocol. Each attempt was conducted under consistent environmental conditions, including identical instrumentation, microscope settings, and operator positioning [1].

In addition to the original protocol guidelines, a grading system was introduced to assess the difficulty level of each placenta specimen [16]. This modification aimed to reduce biological variability between samples by discarding any specimens labeled as "high difficulty," which could introduce additional bias.

The task consisted of three sequential stages. The first involved a 360° circumferential dissection of an artery from the surrounding placental tissue. The second stage required meticulous adventitial plane dissection of the exposed artery. The final stage comprised a microvascular end-to-end anastomosis, executed with five 10-0 nylon stitches placed circumferentially at the free ends of the vessels.

Performance was evaluated at each stage using two core variables: the time required to complete each task stage and a microsurgical error scoring system that categorized technical mistakes. Additionally, at the end of each attempt, a patency test was performed to assess anastomosis unobstructed flow.

An “attempt” is defined as a complete procedure in which the operator successfully finishes all three task stages and achieves the objectives without any exclusion criteria. According to the previous protocol, inclusion required uninterrupted task execution with acceptable performance. Exclusion applied to any attempt that either reached the maximum mistake score or involved procedural interruptions, such as pauses that required the timer to be stopped while transitioning between stages [1]. For the present trial, the inclusion criteria remain the same, except that major mistakes are now recorded and specifically considered in further calculations.

Microsurgical performance assessment and variables

A comprehensive overview of the selected variables, their measurements, and definitions is provided in Table 1.

**Table 1:** Variable definition and interpretation

Variable	Name	Definition	Source	Interpretation
TCT	Time to complete the task	Elapsed time in seconds	Directly registered	The time needed to achieve a goal (improved skill = faster accomplishment)
TMM	Task with a major mistake	Mistake score total >4	Directly registered	The quality of the end-product, measured in mistakes (improved skill = higher quality = less mistakes)
MMA	Major mistake average	TMM/NOA for the same period	Calculated	The relation between the quality evolution in a given period (improved skill = period with diminished mistake tendency)
NOA	Number of attempts	Accumulated attempts in the trial	Directly registered	No independent meaning. It measures the effective time progression

Three principal variables were selected for analysis. The first was the time to complete the task (TCT), which measured the duration (in seconds) required to perform the complete procedure. The second was the mistake score, based on a predefined scoring system that categorized errors as either minor or major. The third variable, the number of attempts (NOA), served as an index of procedural repetition and temporal progression. The error scoring system distinguished between two types of microsurgical mistakes.

1) Minor mistake (1 point): A technical error that did not compromise the final outcome and was potentially repairable (e.g., small leakage during patency testing).

2) Major mistake (5 points): A critical error that prevented successful completion or required major corrective action (e.g., arterial wall tear during dissection).

The mistake score rubric was fully described in the original protocol article [1] and was considered appropriate to assess the procedure's quality. Additional materials have been included in the Appendix, pointing out particular examples for their application.

Repairs were not permitted during the trial, and all errors were recorded. An attempt was classified as involving a "major mistake" if the cumulative mistake score was ≥5. Due to the confounding effects observed with minor errors, often influenced by external factors such as simulator variability or strategy adjustments, major mistakes were prioritized in the analysis as a more reliable indicator of technical performance.

To assess the cumulative quality of performance over time, a novel variable was introduced: the major mistake average (MMA).

\( \text{MMA} = \frac{\text{Number of accumulated attempts with a major mistake in a period}}{\text{Number of all accumulated attempts in the same period}} \)

This index represented the mean occurrence of major mistakes across the attempts and provided a clearer depiction of learning progression. Learning curve phases were delineated based on changes in the MMA and NOA. Notable inflection points were identified and subjected to further statistical scrutiny.

Statistical analysis

All data were collected in accordance with the training protocol. Initial calculations were performed using Google Sheets® (Google, Mountain View, CA) and Microsoft Excel® (Microsoft Corporation, Redmond, WA), followed by database construction and advanced analysis in PostgreSQL® (Global Development Group, USA). This platform enabled seamless integration with algorithms based on Python® (Python Software Foundation, Wilmington, DE) for observation and statistical verification.

A time-series model was constructed using an autoregressive integrated moving average (ARIMA) approach. The model's goodness of fit was evaluated using the coefficient of determination (R²).

To assess the randomness or predictability of error occurrence in the latter stages of the trial, the Poisson dispersion test was applied. This test is designed to determine whether discrete events, in this case, major mistakes, occur randomly or follow a nonrandom pattern, suggesting underlying causative factors. A positive result (i.e., acceptance of the null hypothesis) indicates that the event follows a Poisson distribution and thus arises randomly. In contrast, a negative result suggests that the event is systematically influenced, such as by operator inexperience or protocol complexity.

In the context of surgical simulation, confirmation of Poisson distribution implies that the operator has achieved a level of technical skill sufficient to perform the procedure reliably and that residual errors are likely due to chance rather than skill deficits. This test was, therefore, used to validate the endpoint of the learning curve, identifying when technical performance reached a plateau indicative of an effective skill level.

## Results

The study was conducted from April 2024 to January 2025 and included a total of 132 end-to-end anastomosis attempts performed over 65 sessions. Each session included approximately four attempts, with an average interval of four days between the sessions (range: 1-35 days). Table 2 presents a sample of the raw data, including all variables and their respective measurements. Each stage of the task has individual records for time and mistakes. There was no need to exclude any procedure at the end of the trial.

**Table 2:** Raw data sample Partial sample of 18 records from the full table for all 132 attempts. The full table is available in the Appendix due to its extent

Attempt	Date	Mistakes				Time			
		Stage1	Stage2	Stage3	Total score	Stage1	Stage2	Stage3	Total time
1	April 18, 2024	-	-	1	1	776	203	1,159	2,138
2	April 19, 2024	-	1	1	2	1,002	859	752	2,613
3	April 18, 2024	-	-	-	0	408	562	901	1,871
4	April 20, 2024	-	1	-	0	484	648	1,925	3,057
5	April 24, 2024	-	-	-	0	619	452	1,865	2,936
6	April 24, 2024	-	5	-	5	-	-	-	0
7	April 25, 2024	-	-	1	1	690	646	1,458	2,794
8	April 24, 2024	-	-	1	1	404	397	840	1,641
9	April 26, 2024	-	-	2	1	435	135	1,028	1,598
10	April 26, 2024	-	-	-	0	446	946	690	2,082
11	April 26, 2024	-	-	-	0	519	593	822	1,934
12	April 29, 2024	-	-	-	1	246	505	1,188	1,939
13	April 30, 2024	5	-	-	5	-	-	-	0
14	April 30, 2024	-	5	-	5	-	-	-	0
15	April 30, 2024	-	-	-	0	472	508	962	1,942
16	May 2, 2024	-	5	-	5	-	-	-	0
17	May 2, 2024	-	1	1	2	711	648	1,079	2,438
18	May 2, 2024	-	1	-	1	366	573	1,039	1,978

Time to complete the task 

**Figure 1:**
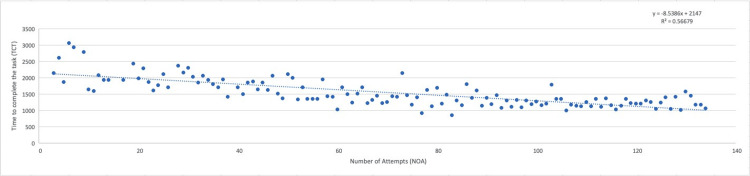
Time to complete the task dispersion and trend The dotted line indicates a calculated linear trend (R^2 ^= 0.566)

The cumulative microscope time totaled 53 hours, 59 minutes, and 25 seconds. The longest recorded task duration occurred during the fourth attempt, lasting 3,057 seconds, while the shortest was observed at the 81st attempt, with a duration of 860 seconds. The mean time to complete the task (TCT) was 1,495.11 seconds, with a median of 1,413 seconds. Although the TCT demonstrated a general downward trend throughout the training period, it also exhibited significant variability, resulting in a coefficient of determination (R²) of 0.566. These data are illustrated in Figure 1.

Major mistakes

**Figure 2:**
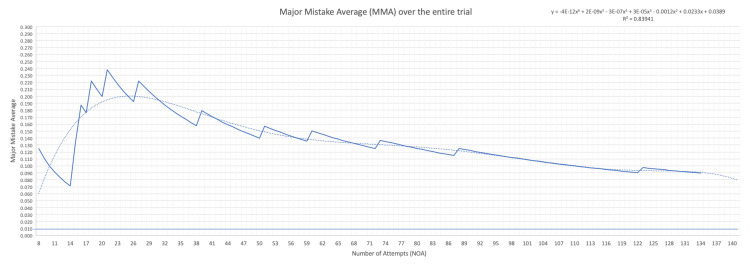
Major mistakes average in chronological ordered of attempts Dotted line showing exponentially calculated trend line (R^2^ = 0.839)

Across all attempts, 11 major mistakes were documented. Of these, eight occurred during Stage 2 (adventitial dissection), two during Stage 1 (circumferential arterial dissection), and one during Stage 3 (suturing). Notably, no attempt was classified as a “major error” due solely to the accumulation of minor mistakes. The first major mistake occurred during the sixth attempt, yielding an MMA of 0.167. The MMA peaked at 0.263 by the 19th attempt and subsequently declined, reaching its lowest recorded value of 0.091 at the 132nd attempt. The learning curve constructed using MMA as the primary outcome variable demonstrated a consistent downward trajectory with a strong correlation (R² = 0.839), as shown in Figure 2.

Based on the MMA trend, six distinct periods were identified within the learning curve. During the initial five attempts, no major mistakes were observed, and thus, the MMA could not be calculated. The subsequent period, encompassing attempts 6-12, demonstrated a transient improvement followed by a rapid increase in MMA. Similarly, the third period (attempts 13-19) culminated in the highest MMA value, indicating pronounced fluctuation and a lack of predictive utility. These early phases were considered unstable and likely influenced by insufficient data. The fourth period, spanning attempts 20-36, exhibited a steady decline in MMA from 0.263 to 0.167, with an absolute drop of 0.096 and a calculated drop rate of 0.006. The fifth period, between attempts 37 and 85, demonstrated a more gradual improvement, as the MMA decreased to 0.118, with an absolute drop of 0.049 and a drop rate of 0.001. Finally, during the sixth and final period (attempts 86-132), the MMA stabilized at 0.091, with a minimal drop rate of 0.0005, indicating a plateau in skill acquisition.

Poisson dispersion test

To further assess the nature of error occurrence during the later phases of training, the Poisson dispersion test was applied to periods 4-6. While the first two of these periods did not meet the criteria for Poisson conformity, the final period (attempts 86-132) did, with a chi-square value of 3.841. This finding supports the interpretation that major mistakes during this phase occurred randomly and were no longer primarily attributable to technical insufficiencies. A summary of the learning curve phases and corresponding statistical data is provided in Table 3.

**Table 3:** Main findings at the learning curve of this trial The TCT values are taken as an average and shown in the column TCTa LC: learning curve; NOA: number of attempts; MMA: major mistake average; TCT: task completion time average (average value for all TCT in the corresponding period); N/A: not available

LC period	Range	NOA	MMA	Drop rate	Interpretation	TCTa	Poisson	Control
1	Attempts 1-5	5	N/A	N/A	Not major mistakes committed yet	2,523.00	N/A	N/A
2	Attempts 6-12	7	N/A (false 0.083)	N/A	Not enough data to calculate	1,712.60	N/A	N/A
3	Attempts 13-19	7	N/A (base 0.263)	N/A	Not enough data to calculate	1,236.30	N/A	N/A
4	Attempts 20-36	17	0.167	0.006	Effective change in skill, mistakes still depend on the operators' skill	1,805.88	Negative	Lambda = 0.0625; Chi^2^ = 9.487
5	Attempts 37-85	49	0.118	0.001	Effective change in skill, mistakes still depend on the operators' skill	1,469.92	Negative	Lambda = 0.0819; Chi^2^ = 7.814
6	Attempts 86-132	47	0.091	0.0005	Effective change in skill, mistakes depending on a random pattern	1,237.22	Positive	Lambda = 0.01; Chi^2^ = 3.841

## Discussion

At the outset of our research and review, we identified substantial and previously published evidence on related subtopics.

Conceptual framework and literature review 

At the outset of this investigation, a comprehensive review of the literature revealed a substantial body of evidence addressing microsurgical skill acquisition, learning curve assessment, and simulation-based training. To establish a clear and consistent framework for this study, the term skill was defined as the capacity to perform a task with consistent dexterity and efficiency within a specific technical domain, with potential progression toward full competency.

Previous meta-analyses have emphasized the importance of measuring both the quantity and quality of movement as central components of surgical skill evaluation [2]. Indirect measurement of movement quantity can be achieved through metrics such as task completion time, while direct assessments may employ tools like motion tracking, video analysis, or wearable sensors [17-19]. However, evaluating movement quality remains inherently more challenging, requiring robust scoring systems and/or other complex methods of evaluation.

Simulation models can be broadly categorized based on their training objectives. Those designed for general skills emphasize interaction with realistic biological or synthetic tissue. In contrast, task-specific simulators often incorporate defined anatomic features, surgical environments, and haptic fidelity tailored to specific procedures [20-22]. In the present study, the chosen biological simulator (human placenta) enabled realistic tissue handling and microdissection while remaining accessible and cost-effective. Proper technical instruction prior to simulation is critical for ensuring reliable data. The operator in this study underwent standardized training in micromovement techniques and was familiarized with the protocol through guided instruction and review of pertinent literature. This preparation helped minimize learning variability unrelated to procedural repetition [23,24].

As data science continues to advance, the application of artificial intelligence and algorithmic modeling to surgical education has gained momentum [7-9]. While these innovations are promising, recent bibliometric analyses indicate that traditional approaches, such as structured protocols and objective scoring, remain among the most trusted methods for skill assessment [6]. Moreover, emerging technologies must be evaluated not only for accuracy but also for feasibility, cost, and educational impact. In this regard, the present study sought to balance technological rigor with practical applicability. The challenges inherent in skill evaluation are well-documented. These include interoperator variability, limited simulator standardization, subjective scoring (which is significantly increased by a non-blinded evaluation), and the absence of a universally accepted benchmark for technical proficiency. The present study acknowledged these complexities and aimed to address them through a combination of standardized training, consistent data acquisition, an objective approach, and a statistically validated performance index [11,25,26].

Study findings 

Analysis of the collected dataset highlighted key distinctions between the two primary performance indicators: TCT and MMA. Although TCT displayed a general downward trend over the course of training, it was found to be significantly influenced by external variables, such as intersession intervals and inherent differences in the biological simulator. For instance, attempts performed after extended breaks tended to be slower and more error-prone, while intrasession repetitions often demonstrated improved speed. Additionally, excessive acceleration during plateau-phase attempts was occasionally associated with an increased risk of error, suggesting that rapid performance may compromise precision even in experienced operators.

In contrast, the MMA emerged as a more robust and consistent indicator of technical skill. This metric captured the frequency of critical, nonrepairable errors, events that are less likely to be affected by minor procedural variability. When MMA values were plotted over the number of attempts, a characteristic learning curve emerged, displaying an initial phase of instability, followed by progressive improvement, and finally a plateau consistent with skill stabilization.

The identification of distinct learning curve (LC) periods, each defined by changes in MMA slope, allowed for nuanced interpretation of the operator’s progression. Early LC periods (attempts 6-19) were marked by substantial variability, likely reflecting an insufficient volume of data and inconsistent performance. In contrast, subsequent LC periods (attempts 20-85) showed a clear trend toward stabilization, with decreasing mistake frequency and reduced slope. Notably, the final LC period (attempts 86-132) demonstrated sustained low MMA values and a minimal drop rate, suggesting that the operator had reached a level of technical maturity.

Application of Poisson dispersion theory to surgical skill assessment

To further validate this interpretation, Poisson dispersion testing was employed to assess whether the occurrence of major mistakes in the later LC periods could be attributed to random variability. This theory has a proven track record in measuring random events and has been widely used [27,28], including healthcare-related publications [29,30]. The underlying premise is that, once a sufficient level of skill is achieved, errors no longer occur due to technical inadequacy but rather as stochastic events intrinsic to any complex task. In this framework, a Poisson-positive result indicates that mistakes are randomly distributed, supporting the conclusion that the operator has reached a plateau of competence.

**Figure 3:**
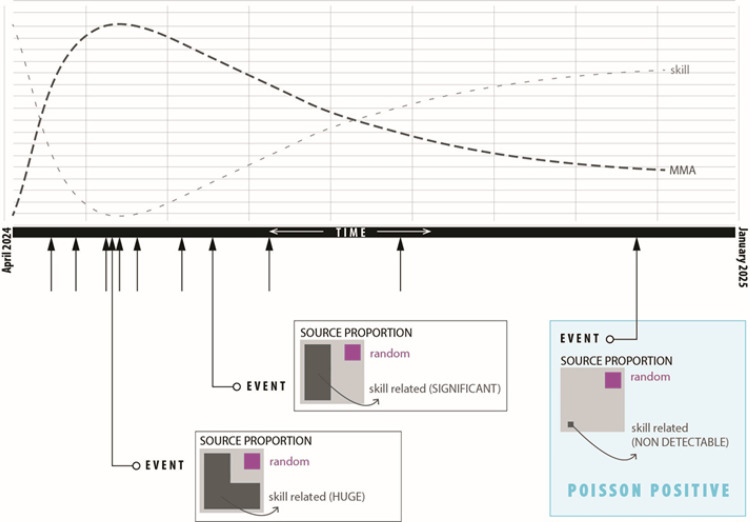
Poisson’s dispersion theory interpretation diagram The black dashed line summarizes the evolution of the major mistake average index. The gray dashed line represents theoretical skill evolution. Arrows pointing upward on the baseline represent the occurrence of each major mistake along the trial. Boxes below the baseline indicate the interpretations at each corresponding point Image credit: This is an original image created by the author Pablo J. Villanueva

Indeed, the final LC period met the criteria for Poisson conformity (Chi² = 3.841), suggesting that remaining errors were not systematically associated with skill deficiencies. This finding supports the novel proposal that Poisson theory may be used to objectively signal the attainment of an “efficient skill threshold” in surgical training. To the best of our knowledge, this represents a unique application of Poisson modeling in the assessment of microsurgical performance (Figure 3).

Future direction and limitations

The implications of these findings extend beyond this individual trial. The MMA-based learning curve, corroborated by Poisson validation, offers a replicable and cost-effective framework for assessing skill acquisition across various surgical domains. It also opens avenues for defining objective, data-driven endpoints for microsurgical training programs, where the transition from systematic error to random error may signify readiness for clinical application.

While promising, these conclusions must be interpreted with caution. The single-operator design limits generalizability, and further studies involving multiple trainees are needed to confirm the reliability and applicability of this method across different individuals and settings.

## Conclusions

This study sought to objectively characterize the microsurgical learning curve by analyzing performance metrics derived from a standardized simulation protocol. Among the two primary variables assessed, the time to complete the task (TCT) demonstrated a modest correlation with skill acquisition but was found to be susceptible to confounding influences such as biological variability and session timing. As a result, it was determined to have limited reliability as a stand-alone indicator of technical progression. In contrast, the major mistake average (MMA) proved to be a stable and independent measure of procedural quality. It effectively captured the operator’s progression through distinct phases of learning and was instrumental in delineating the point at which technical performance plateaued.

A key innovation of this study was the application of Poisson dispersion theory to the final phase of training. This statistical model enabled the differentiation between errors arising from residual technical deficits and those attributable to random variability. The acceptance of a Poisson distribution during the final learning period provided compelling evidence that the operator had achieved a level of proficiency where further mistakes occurred randomly, rather than systematically. This finding introduces a novel and objective method for defining “sufficient expertise” within the context of surgical skill acquisition. Overall, the integration of MMA with Poisson analysis presents a promising framework for assessing microsurgical competence. Future investigations involving multiple operators and diverse surgical tasks will be essential to validate and expand upon these results.

## Appendices

**Table 4:** Mistake score details and examples All possible mistakes, as used in the original protocol, are summarized

Mistake abbreviation	Name	Value	Description
M	Major mistake	5 points	The selected vessel for dissection is damaged (bleeding from a wall break). Sutured vessel appears nonpermeable during testing. Patency verification tests show nonflux or nonsignificant flux through the suture line
m	Minor mistake	1 point	Small branches coming from the parental vessel are cut, intentionally or not, instead of being avoided by fine dissection. Laminar chorionic membrane, the deeper layer under the vessel bottom, is breached, leading to bleeding from the decidua levels. Sutured vessel shows a leak along the suture line (either between stitches or due to a stitch coming loose during the patency test)

**Table 5:** Full raw data

Attempt	Date	Mistake				Time			
		Stage 1	Stage 2	Stage 3	Total mistake	Artery 1	Adventitia	Points 1-4	Total
1	April 18, 2024	-	-	1	1	776	203	1,159	2,138
2	April 19, 2024	-	1	1	2	1,002	859	752	2,613
3	April 19, 2024	-	-	-	0	408	562	901	1,871
4	April 20, 2024	-	1	-	0	484	648	1,925	3,057
5	April 24, 2024	-	-	-	0	619	452	1,865	2,936
6	April 24, 2024	-	5	-	5	-	-	-	0
7	April 25, 2024	-	-	1	1	690	646	1,458	2,794
8	April 25, 2024	-	-	1	1	404	397	840	1,641
9	April 26, 2024	-	-	2	1	435	135	1,028	1,598
10	April 26, 2024	-	-	-	0	446	946	690	2,082
11	April 26, 2024	-	-	-	0	519	593	822	1,934
12	April 29, 2024	-	-	-	1	246	505	1,188	1,939
13	April 30, 2024	5	-	-	5	-	-	-	0
14	April 30, 2024	-	5	-	5	-	-	-	0
15	April 30, 2024	-	-	-	0	472	508	962	1,942
16	May 2, 2024	-	5	-	5	-	-	-	0
17	May 2, 2024	-	1	1	2	711	648	1,079	2,438
18	May 2, 2024	-	1	-	1	366	573	1,039	1,978
19	May 8, 2024	-	1	5	6	474	599	1,223	2,296
20	May 8, 2024	-	1	-	1	444	403	1,020	1,867
21	May 8, 2024	-	-	1	1	292	338	989	1,619
22	May 8, 2024	-	-	-	0	648	473	646	1,767
23	May 9, 2024	-	-	1	1	484	308	1,321	2,113
24	May 9, 2024	-	-	-	0	483	273	957	1,713
25	May 22, 2024	-	5	-	5	-	-	-	0
26	May 22, 2024	-	-	2	2	477	423	1,473	2,373
27	May 23, 2024	-	-	2	2	384	460	1,320	2,164
28	May 26, 2024	-	-	-	0	288	548	1,478	2,314
29	May 26, 2024	-	-	-	0	426	746	856	2,028
30	May 28, 2024	-	-	1	1	712	194	947	1,853
31	May 28, 2024	-	-	1	1	791	131	1,150	2,072
32	May 28, 2024	-	-	-	0	480	240	1,216	1,936
33	May 29, 2024	-	-	1	1	514	302	988	1,804
34	May 29, 2024	-	-	1	1	481	458	776	1,715
35	May 30, 2024	-	-	1	1	253	496	1,200	1,949
36	May 30, 2024	-	1	1	2	422	340	651	1,413
37	May 30, 2024	-	5	-	5	-	-	-	0
38	May 30, 2024	-	-	1	1	260	387	1,055	1,702
39	May 30, 2024	-	-	1	1	203	170	1,127	1,500
40	May 31, 2024	-	-	1	1	745	242	869	1,856
41	June 6, 2024	-	-	1	1	432	619	838	1,889
42	June 7, 2024	-	-	-	0	298	256	1,086	1,640
43	June 12, 2024	-	-	1	1	458	365	1,037	1,860
44	June 12, 2024	-	-	1	1	303	379	942	1,624
45	June 13, 2024	-	-	1	1	489	620	948	2,057
46	June 13, 2024	-	-	-	0	321	240	956	1,517
47	June 14, 2024	-	-	1	1	299	282	797	1,378
48	June 28, 2024	-	-	1	2	370	405	1,332	2,107
49	July 10, 2024	-	5	1	6	473	190	1,343	2,006
50	July 10, 2024	-	-	1	2	301	115	927	1,343
51	July 11, 2024	-	-	1	1	422	284	1,009	1,715
52	July 11, 2024	-	-	-	0	196	183	971	1,350
53	July 12, 2024	-	-	-	0	380	194	777	1,351
54	July 12, 2024	-	-	-	0	301	277	769	1,347
55	July 14, 2024	-	-	-	0	356	506	1,097	1,959
56	July 14, 2024	-	-	-	0	285	279	876	1,440
57	July 15, 2024	-	-	-	0	404	376	643	1,423
58	July 17, 2024	-	5	1	6	230	269	525	1,024
59	July 17, 2024	-	-	1	1	253	213	1,249	1,715
60	July 18, 2024	-	-	1	1	328	375	793	1,496
61	July 18, 2024	-	-	-	0	281	167	798	1,246
62	July 19, 2024	-	-	-	0	343	416	761	1,520
63	July 25, 2024	-	-	1	1	481	150	1,082	1,713
64	July 25, 2024	-	-	-	1	366	256	606	1,228
65	July 25, 2024	-	-	-	0	278	223	825	1,326
66	July 26, 2024	-	-	1	1	266	253	935	1,454
67	July 26, 2024	-	-	-	0	231	298	688	1,217
68	July 26, 2024	-	-	1	1	220	411	630	1,261
69	July 26, 2024	-	-	1	1	226	277	932	1,435
70	July 26, 2024	-	-	-	0	189	549	677	1,415
71	August 29, 2024	5	-	-	5	413	525	1,207	2,145
72	August 29, 2024	-	-	-	0	530	264	676	1,470
73	August 29, 2024	-	-	-	0	224	370	582	1,176
74	September 4, 2024	-	-	2	2	326	385	692	1,403
75	September 4, 2024	-	-	-	0	204	138	573	915
76	September 5, 2024	-	-	-	0	302	442	882	1,626
77	September 5, 2024	-	-	1	1	270	225	637	1,132
78	September 6, 2024	-	-	1	1	306	376	1,010	1,692
79	September 6, 2024	-	-	-	0	200	238	773	1,211
80	September 11, 2024	-	-	-	0	298	440	740	1,478
81	September 11, 2024	-	-	-	1	203	159	498	860
82	September 12, 2024	-	-	1	1	310	378	614	1,302
83	September 12, 2024	-	-	-	0	243	331	594	1,168
84	September 13, 2024	-	-	-	0	243	572	995	1,810
85	September 13, 2024	-	-	-	0	301	238	845	1,384
86	September 25, 2024	-	5	-	5	230	289	1,091	1,610
87	September 25, 2024	-	-	-	0	131	192	824	1,147
88	September 26, 2024	-	-	1	1	225	278	885	1,388
89	September 26, 2024	-	-	-	0	185	346	661	1,192
90	September 27, 2024	-	-	1	0	249	194	1,016	1,459
91	September 27, 2024	-	-	-	0	123	179	776	1,078
92	October 2, 2024	-	-	2	2	384	232	682	1,298
93	October 2, 2024	-	-	1	1	206	210	693	1,109
94	October 3, 2024	-	-	1	1	266	307	743	1,316
95	October 3, 2024	-	-	1	1	330	189	575	1,094
96	October 3, 2024	-	-	-	0	445	140	723	1,308
97	October 3, 2024	-	-	-	0	335	87	769	1,191
98	October 4, 2024	-	-	-	0	214	147	911	1,272
99	October 4, 2024	-	-	1	1	170	281	706	1,157
100	October 10, 2024	-	-	1	1	196	192	827	1,215
101	October 29, 2024	-	-	3	3	270	412	1,103	1,785
102	October 29, 2024	-	-	1	1	353	193	814	1,360
103	October 31, 2024	-	-	-	0	243	319	792	1,354
104	November 1, 2024	-	-	-	0	179	102	718	999
105	November 7, 2024	-	-	-	0	231	230	723	1,184
106	November 8, 2024	-	-	1	1	182	230	737	1,149
107	November 22, 2024	-	-	1	1	237	328	565	1,130
108	November 22, 2024	-	-	-	0	200	297	760	1,257
109	November 22, 2024	-	-	-	0	280	85	771	1,136
110	December 3, 2024	-	-	2	2	279	249	832	1,360
111	December 3, 2024	-	-	1	2	269	57	784	1,110
112	December 3, 2024	-	-	1	2	156	224	988	1,368
113	December 3, 2024	-	-	1	2	184	111	858	1,153
114	December 3, 2024	-	-	-	0	213	145	678	1,036
115	December 5, 2024	-	-	-	0	156	211	783	1,150
116	December 7, 2024	-	-	1	1	184	284	878	1,346
117	December 7, 2024	-	-	-	0	230	205	797	1,232
118	December 7, 2024	-	-	-	0	183	292	730	1,205
119	December 7, 2024	-	-	1	1	283	192	728	1,203
120	December 6, 2024	-	-	1	1	261	235	805	1,301
121	December 11, 2024	-	5	-	5	265	279	713	1,257
122	December 11, 2024	-	-	-	0	222	302	525	1,049
123	December 12, 2024	-	-	-	0	298	276	661	1,235
124	December 13, 2024	-	-	1	1	273	208	922	1,403
125	December 13, 2024	-	-	-	0	250	196	597	1,043
126	January 4, 2025	-	-	1	1	424	282	718	1,424
127	January 5, 2025	-	-	-	0	186	179	651	1,016
128	January 7, 2025	-	-	-	0	243	241	1,101	1,585
129	January 7, 2025	-	-	1	1	230	315	909	1,454
130	January 7, 2025	-	-	1	1	291	206	672	1,169
131	January 8, 2025	-	-	1	1	217	282	674	1,173
132	January 8, 2025	-	-	-	0	252	157	653	1,062
